# ZnT8 Haploinsufficiency Impacts MIN6 Cell Zinc Content and β-Cell Phenotype via ZIP-ZnT8 Coregulation

**DOI:** 10.3390/ijms20215485

**Published:** 2019-11-04

**Authors:** Rebecca Lawson, Wolfgang Maret, Christer Hogstrand

**Affiliations:** Metal Metabolism Group, Department of Nutritional Sciences, Faculty of Life Sciences and Medicine, King’s College London, 150 Stamford St, London SE1 9NH, UK; beckyy93@hotmail.co.uk (R.L.); wolfgang.maret@kcl.ac.uk (W.M.)

**Keywords:** ZnT8, ZIP, zinc, MIN6 cells, β-cells, Type 2 Diabetes

## Abstract

The zinc transporter ZnT8 (*SLC30A8*) localises to insulin secretory granules of β-cells where it facilitates zinc uptake for insulin crystallisation. ZnT8 abundance has been linked to β-cell survival and functional phenotype. However, the consequences of ZnT8 haploinsufficiency for β-cell zinc trafficking and function remain unclear. Since investigations in human populations have shown *SLC30A8* truncating polymorphisms to decrease the risk of developing Type 2 Diabetes, we hypothesised that ZnT8 haploinsufficiency would improve β-cell function and maintain the endocrine phenotype. We used CRISPR/Cas9 technology to generate ZnT8 haploinsufficient mouse MIN6 β-cells and showed that ZnT8 haploinsufficiency is associated with downregulation of mRNAs for *Slc39a8* and *Slc39a14*, which encode for the zinc importers, Znt- and Irt-related proteins 8 (ZIP8) and 14 (ZIP14), and with lowered total cellular zinc content. ZnT8 haploinsufficiency disrupts expression of a distinct array of important β-cell markers, decreases cellular proliferation via mitogen-activated protein (MAP) kinase cascades and downregulates insulin gene expression. Thus, ZnT8 cooperates with zinc importers of the ZIP family to maintain β-cell zinc homeostasis. In contrast to the hypothesis, lowered ZnT8 expression reduces MIN6 cell survival by affecting zinc-dependent transcription factors that control the β-cell phenotype.

## 1. Introduction

In mammals, the 14 Znt- and Irt-related proteins (ZIP) transport zinc or other metal ions into the cytosol of cells, and cytosolic zinc efflux is mediated by proteins of the zinc transporter (ZnT) family, of which most mammals have 10 paralogues [1]. Approximately 50% of cellular zinc is distributed to the cytosol, where it is buffered in such a way that the “free” available zinc concentrations are about 0.4 nM in rat INS-1E β-cells [2]. Zinc can be involved in cellular signalling and is otherwise tightly bound to metalloproteins as a structural or catalytic cofactor [3]. The zinc transporter ZnT8 highly expressed in β-cells localises to the membrane of insulin secretory granules where it regulates granule zinc uptake and accumulation, important for proper insulin maturation, storage and secretion [4]. ZnT8 expression is associated with altered β-cell function and ZnT8 polymorphisms and is linked to Type 2 Diabetes risk [5,6,7,8]. 

The role of ZnT8 in β-cell function has been extensively explored [4], although the details how the common tryptophan/arginine (W/R) variants affect the Type 2 diabetes risk remain unresolved. Decreased ZnT8 expression impacts β-cell function and mass as observed in hypoxia [9], lipotoxicity [10] and cytokine stimulation [11]. ZnT8 overexpression, on the other hand, protects against palmitate- and zinc chelating agent (TPEN)-induced decreases in glucose-stimulated insulin secretion (GSIS) in human islets [12] and increases the cellular zinc content and protects against zinc depletion-induced apoptosis in INS-1E cells [13]. In another study, it was found that ZnT8 is upregulated in islets of diabetic and insulin-resistant patients. Knockdown in mouse MIN6 β-cells increases proliferation and protects against inflammation-induced cell death [8]. Consistent with an association between ZnT8 and Type 2 Diabetes risk, ZnT8 haploinsufficiency through any of twelve rare loss-of-function nonsense and missense mutations is protective against the disease [14]. However, the effect of an absolute loss of function in humans remains unknown. Islets from ZnT8^+/+^ mice containing knock-in mutations of the most common loss-of-function variant with a pre-mature stop codon following arginine 138 (R138X) show reduced ZnT8 activity coupled with increased insulin secretory function [15]. ZnT8 knockout in the mouse induces large changes in secretory granule morphologies and lowers β-cell zinc content. However, few differences in glycaemic parameters were noted [16,17,18,19]. 

The polymorphic variant rs13266634 (R325W) in the human ZnT8 gene *SLC30A8* is strongly associated with Type 2 Diabetes risk [5,6,7]. Curiously, the R risk allele appears to be associated with a better ability to transport zinc dependent on the lipid composition of the membrane [20]. The C-terminal domain (CTD) of ZnT8-R, which harbours the polymorphism, exhibits a higher thermostability but lower dimerization affinity compared with ZnT8-W, suggesting the mutation impacts transporter stability or sensing [21]. There is no difference in zinc transport activity between the two variants, but the ZnT8-R risk variant is more active in zinc transport than ZnT8-W when the lipid composition is altered in artificial liposomes [20,22]. In MIN6 cells, overexpression of ZnT8-R promotes granule zinc accumulation [16]. Likewise, mouse islets transgenic for human ZnT8-W exhibit reduced zinc content compared to human ZnT8-R controls [23]. Human ZnT8-R variant carriers present with increased proinsulin:insulin ratios [24], lowered β-cell function [25] and impaired insulin secretion during intravenous glucose tolerance tests [7,26]. Because these results have not clarified the exact role of the mutation in developing diabetes, further investigations of the consequences of ZnT8 expression for β-cell zinc metabolism and function are warranted.

We have previously shown that prolonged stimulation of insulin secretion, mimicking the stress β-cells experience in pre-diabetes, decreases the total zinc content of MIN6 cells and changes the expression of genes involved in cellular zinc regulation [27]. Since ZnT8 abundance is linked to β-cell survival and function [8,9,11,12,13,14] and since increased abundance during cellular stimulation elevates cytosolic free Zn^2+^ [28], ZnT8 is likely central to β-cell zinc trafficking and homeostasis. Here, we postulated that ZnT8 functions cooperatively with ZIP transporters. To address this hypothesis and to investigate potential mechanisms of protection against Type 2 Diabetes in ZnT8 haploinsufficient human populations, we knocked down ZnT8 by siRNA and in addition generated and characterised ZnT8 haploinsufficient MIN6 cells. Contrary to what might be expected based on the effect observed in humans with reduced ZnT8 function, the haploinsufficient MIN6 cells exhibited a changed β-cell phenotype in terms of decreased cellular zinc, insulin, and proliferation and lowered survival.

## 2. Results 

### 2.1. ZnT8 Expression Is Associated with Expression of ZIP8 and ZIP14

**Figure 1:**
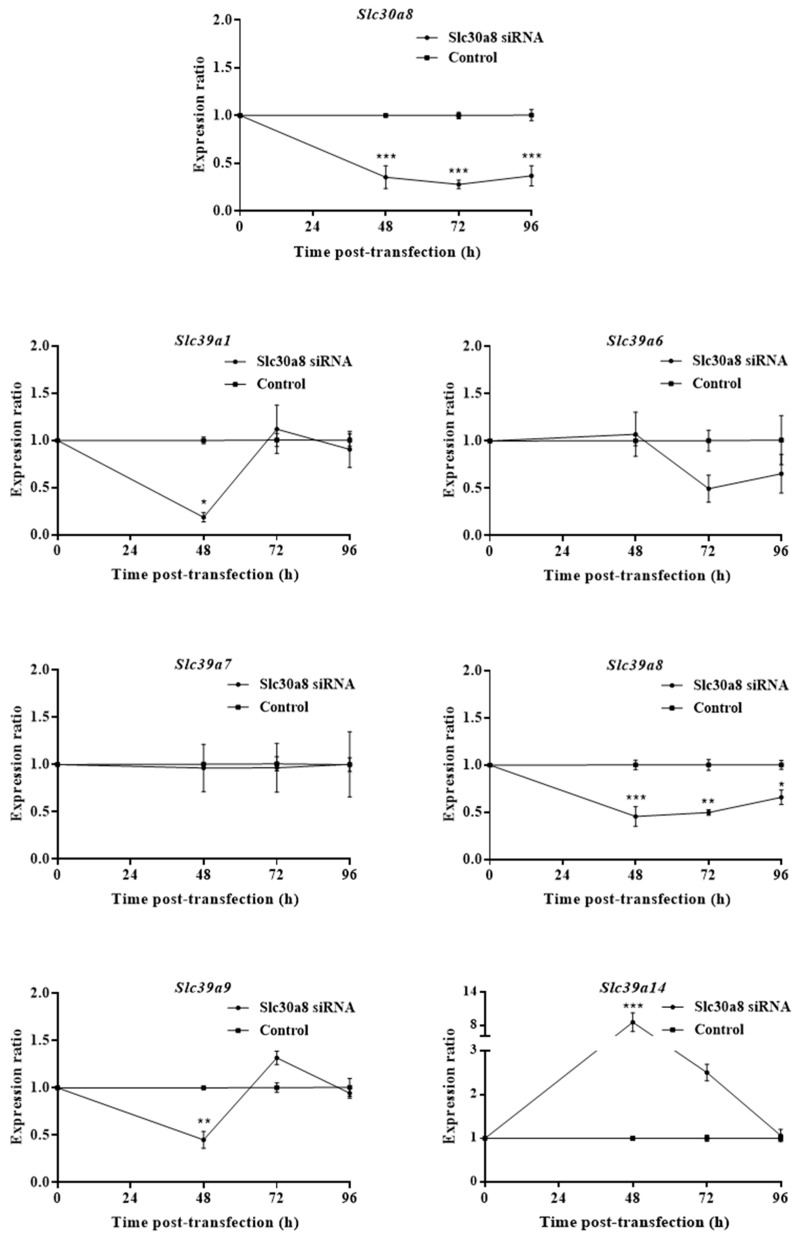
*Slc39a* zinc transporter mRNA expression following transient *Slc30a8* knockdown in MIN6 cells. Time-course for mRNA expression of *Slc30a8, Slc39a1, Slc39a6, Slc39a7, Slc39a8, Slc39a9* and *Slc39a14* following knockdown of *Slc30a8* by siRNA. Expression was assayed at 48, 72 and 96 h post-transfection. Changes in mRNA abundances were calculated through qPCR and presented as expression ratios relative to MIN6 cells transfected with Silencer^®^ Select negative control siRNA (control) at each time-point. Data were analysed by 2-way ANOVA followed by Sidak’s multiple comparison test. Error bars show ±SEM. *n* = 3. * *p* < 0.05, ** *p* < 0.005, *** *p* < 0.001.

To explore the temporal ZIP response as cells adjust to ZnT8 deficiency, we knocked down *Slc30a8* mRNA in MIN6 cells using siRNA and assayed expression of the *Slc39a* paralogues that we and others previously identified as important for β-cell function [28,29,30] at 48, 72 and 96 h (Figure 1). *Slc30a8* showed >2-fold depletion at all three time-points. At 48 h, we observed upregulation of mRNA for *Slc39a14* (8.56-fold), and downregulation for *Slc39a1* (5.39-fold), *Slc39a8* (2.20-fold) and *Slc39a9* (2.22-fold). At both 72 and 96 h, only *Slc39a8* remained differentially expressed compared to control cells (2.02-fold and 1.52-fold downregulation, respectively). These results show that ZnT8 expression affects the expression of ZIP transporters. 

**Figure 2:**
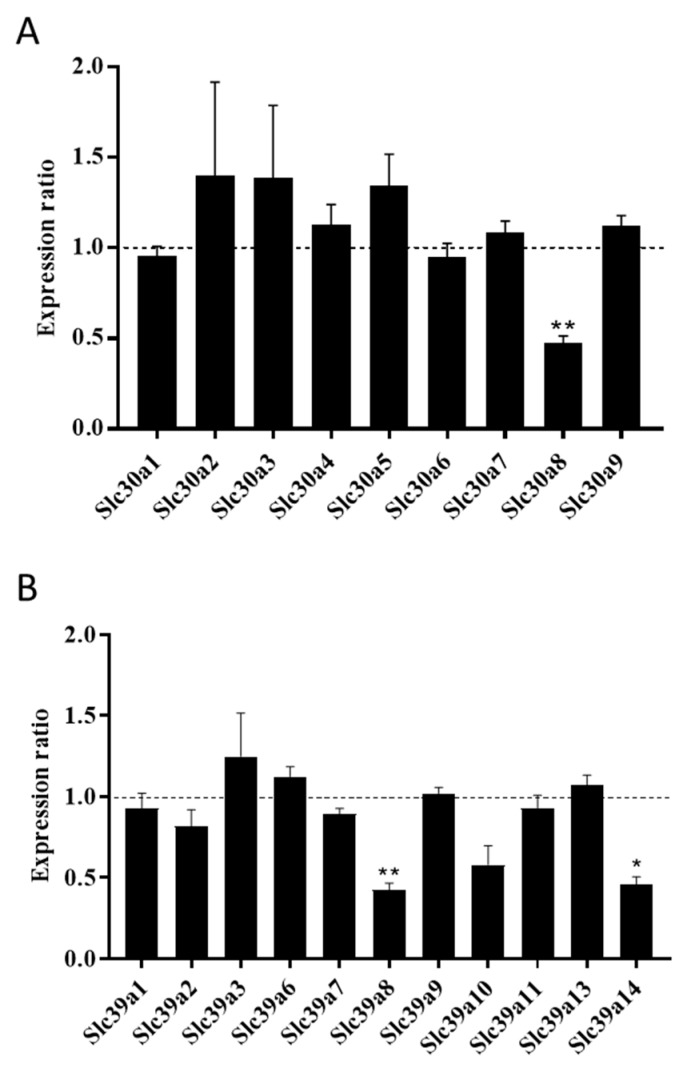
Zinc transporter expression in ZnT8 haploinsufficient MIN6 cells. The graphs show mRNA expression for (**A**) *Slc30a* and (**B**) *Slc39a* paralogues in ZnT8 haploinsufficient MIN6 cells generated by CRISPR/Cas9 technology. We were unable to detect quantifiable expression for *Slc30a10, Slc39a4, Slc39a5* or *Slc39a12*. Changes in mRNA expression were measured by qPCR and are presented as the ratio relative to the expression level in sham-CRISPR MIN6 cells (control), which is indicated by the dotted line. Experiments were carried out at 48 h. Data were analysed through *t*-tests and corrected for multiple comparisons. Error bars show ±SEM. *n* = 3. * *p* < 0.05, ** *p* < 0.005.

We next used CRISPR/Cas9 technology to knock out one of the *Slc30a8* alleles in MIN6 cells (Figure S1) and examined the expression profiles for ZnT and ZIP paralogues in the gene-edited MIN6 cells. In silico sequence analysis predicted that our ZnT8 CRISPR MIN6 cells encode a 187 carboxy-terminal residue-truncated version of ZnT8 in addition to the 367 amino acid wild-type (Figure S2). We observed a 50% reduction of total *Slc30a8* mRNA, suggesting that genome editing either prevented transcription of the truncated *Slc30a8* copy variant or resulted in transcription of unstable, rapidly degraded mRNA, therefore confirming that the gene editing resulted in a ZnT8 haploinsufficient genotype (Figure 2A). We did not detect differences in mRNA abundances for any other ZnT paralogue (Figure 2A), indicating lack of transporter redundancy and/or a compensatory response. When we examined expression of the ZIP paralogues in ZnT8 haploinsufficient MIN6 cells, we recorded downregulation of mRNA for both *Slc39a8* (2.37-fold) and *Slc39a14* (2.32-fold) (Figure 2B).

### 2.2. ZnT8 Haploinsufficiency Decreases Zinc Uptake into MIN6 Cells 

**Figure 3:**
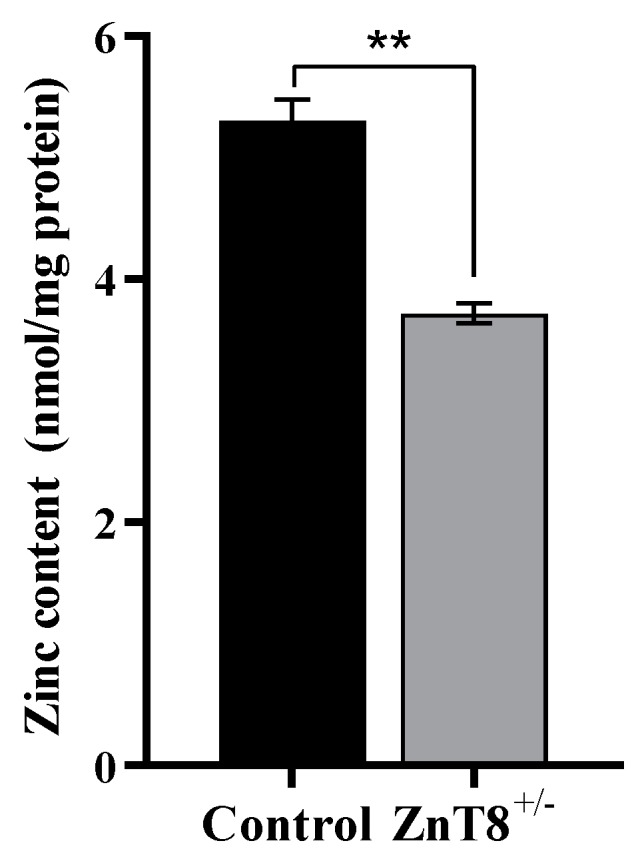
Zinc content of ZnT8 haploinsufficient MIN6 cells. Total cellular zinc content was determined through ICP-MS and normalised to protein. Experiments were carried out at 48 h. Data were analysed by 2-way ANOVA followed by Tukey’s multiple comparison test. *n* = 4. ** *p* < 0.005.

ZnT8 knockout in the mouse decreases the zinc content of islets [16,17,18,19,31,32], indicating ZnT8 affects normal zinc uptake and accumulation. Since ZIP8 and ZIP14 transport iron [33,34] and manganese [35] in addition to zinc, altered ZnT8 expression may also impact the cellular levels of these metal ions. Therefore, we next characterised the impact of ZnT8 haploinsufficiency on the zinc, iron and manganese contents of MIN6 cells. We incubated ZnT8 haploinsufficient MIN6 cells in media containing a normal concentration of zinc (8 μM) for 48 h and found that ZnT8 haploinsufficiency lowered total cellular zinc by 1.43-fold compared with sham-CRISPR MIN6 cells (Figure 3). We did not observe any statistically significant differences in the total cellular contents of iron (ZnT8 haploinsufficient cells: 0.55 nmol/mg; control cells: 0.61 nmol/mg; *p* = 0.95 (*n* = 4)) or manganese (ZnT8 haploinsufficient cells: 0.04 nmol/mg; control cells: 0.06 nmol/mg; *p* = 0.24 (*n* = 4)) at 48 h.

### 2.3. ZnT8 Haploinsufficiency Alters Expression of Important β-Cell Markers

**Figure 4:**
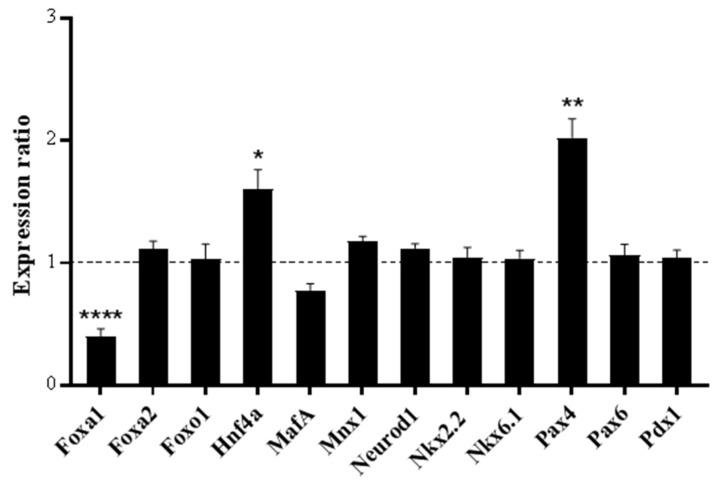
mRNA expression for transcription factors in ZnT8 haploinsufficient MIN6 cells. Expression of each gene was assayed through qPCR and is presented as a ratio compared to the expression level in sham-CRISPR MIN6 cells (control), as indicated by the dotted line. Experiments were carried out at 48 h. Data were analysed through t-tests and corrected for multiple comparisons. Error bars show ±SEM. *n* = 3. * *p* < 0.05, ** *p* < 0.005, **** *p* < 0.0001.

We have previously shown that multiple markers for β-cell identity and function are zinc-responsive (*Hnf1b, Hnf4a, MafA, Mnx-1, Nkx2.2, Nkx6.1*, *Pax4, Pax6* and *Pdx-1*) [27]. Since lowered ZnT8 expression alters β-cell zinc trafficking and reduces total cellular zinc, it was deemed likely that ZnT8 haploinsufficiency impacts expression of these transcription factors. We examined mRNA abundances for the Zn^2+^-responsive transcription factors in ZnT8 haploinsufficient MIN6 cells, in addition to *Foxa1*, *Foxa2*, *Foxo1* and *Neurod1.* We observed altered expression for three β-cell markers in ZnT8 haploinsufficient MIN6 cells (Figure 4), namely, upregulation of mRNA for *Pax4* (2.03-fold) and *Hnf4a* (1.61-fold) and downregulation for *Foxa1* (2.48-fold). 

### 2.4. ZnT8 Haploinsufficiency Reduces MIN6 Cell Proliferation and Ins1 Expression and Secretion

To investigate whether the affected markers for β-cell identity alter the β-cell functional phenotype, we examined the survival parameters and insulin secretory response of ZnT8 haploinsufficient MIN6 cells following culture with normal (8 μM) zinc concentration.

**Figure 5:**
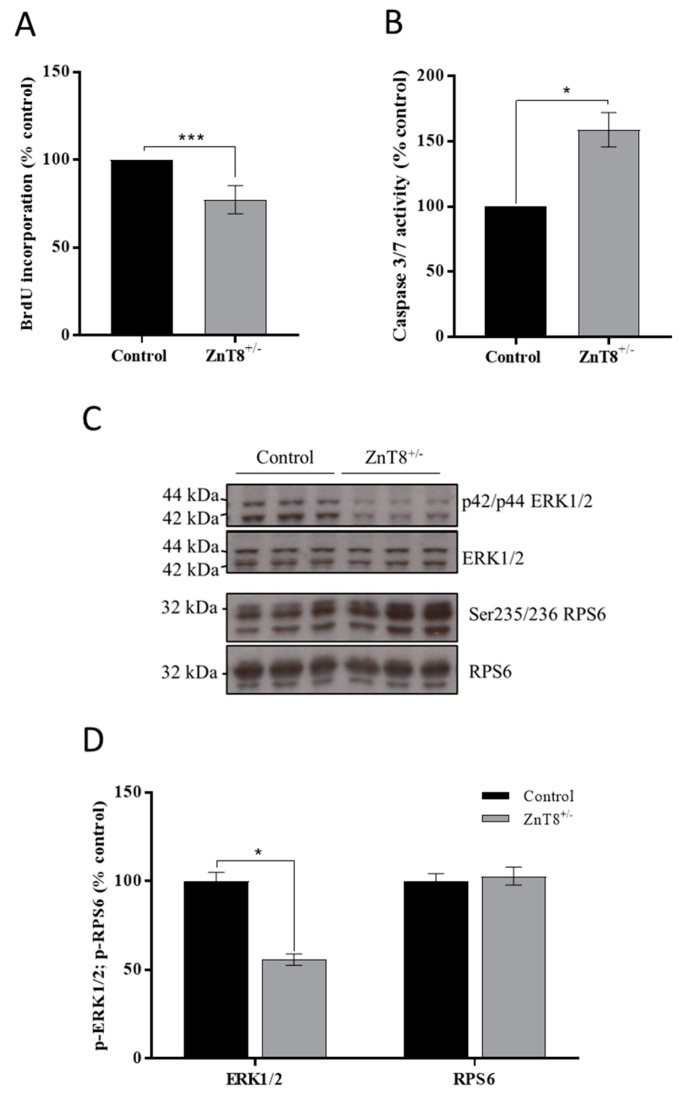
Survival of ZnT8 haploinsufficient MIN6 cells in response to zinc depletion. (**A**) MIN6 cell proliferation, determined by bromodeoxyuridine (BrdU) incorporation. (**B**) MIN6 cell apoptosis, determined by Caspase 3/7 assays. (**C**,**D**) Phosphorylation of ERK1/2 and ribosomal protein S6 (RPS6) shown as (**C**) immunoblots of p42/p44 ERK1/2, ERK1/2, Ser235/236 RPS6 and RPS6 and (**D**) the quantified densities of immunoblot bands for p42/p44 ERK1/2 vs. ERK1/2, and Ser235/236 RPS6 vs. RPS6. Immunoblot quantification was carried out using ImageJ software. All experiments were carried out at 48 h. (**A**,**B**,**D**) Results are relative to sham-CRISPR MIN6 cells (control). Data were analysed by 2-way ANOVA followed by Sidak’s multiple comparison test. ZnT8 haploinsufficient MIN6 cells: ZnT8^+/−^. Error bars show ±SEM. *n* = 3. * *p* < 0.05, *** *p* < 0.001.

ZnT8 haploinsufficient MIN6 cells proliferate less (1.30-fold) compared with sham-CRISPR controls at 48 h. We additionally observed a 1.59-fold increase in apoptosis (Figure 5A,B). Mitogen-activated protein kinase (MAPK) cascades mediate proliferation through cell cycle regulation [36] and mechanistic target of rapamycin (mTOR) cascades promote proliferation through mediating mRNA translation [37]; both MAPK and mTOR pathways can be affected by zinc [38]. We therefore examined MAPK or mTOR signalling cascades by measuring phosphorylation of the downstream effectors ERK1/2 (Mapk1/3) and RPS6 (mTOR), which are both activated by phosphorylation. We observed decreased phosphorylation of ERK1/2 at p42/p44 compared to sham-CRISPR MIN6 cells (1.79-fold) but no effect on RPS6 (Figure 5C–D). 

Finally, we explored the impact of ZnT8 haploinsufficiency on insulin expression and secretion, the downregulation of which is a hallmark of β-cell dysfunction and failure. Unlike in humans, rodents encode two insulin genes, *Ins1* and *Ins2* [39]. We observed 1.65-fold downregulation of *Ins1* mRNA in ZnT8 haploinsufficient MIN6 (*p* < 0.05, *n* = 3) compared with control cells when cultured for 48 h with 8 µM zinc. However, ZnT8 haploinsufficient cells showed no change in abundance of *Ins2* mRNA at either zinc concentration compared with the control (p > 0.05, *n* = 3).

## 3. Discussion

Complete loss of ZnT8 has not been reported in humans. ZnT8 haploinsufficient cells therefore represent a model to examine haploid loss-of-function ZnT8 genotypes [14] and/or ZnT8 downregulation in response to stresses that negatively impact β-cell function and mass [9,11,12]. 

### 3.1. Characteristics of Zinc Metabolism in ZnT8 Haploinsufficient MIN6 Cells 

Upon GSIS, β-cells increase their cytosolic free Zn^2+^ to approximately 850 pM, presumably to increase zinc trafficking to insulin granules [28]; the activity of granule-localised ZnT8 may therefore be coordinated with ZIP-facilitated cytosolic zinc influx at the plasma membrane and/or membranes of intracellular zinc stores [40]. Concurrently, temporal loss of ZnT8 expression induced during β-cell dysfunction is coupled with decreased cytosolic free Zn^2+^ and a reduced insulin secretory capacity [9,11,12]. The lowered cytosolic Zn^2+^ is probably mediated by rapid changes to ZIP function at the plasma membrane and/or other activities coordinated with ZnT8. Following ZnT8 depletion with siRNA, we observed initial transcriptional upregulation of *Slc39a14* and downregulation of *Slc39a1*, *Slc39a8* and *Slc39a9*. Expression of ZIP1, ZIP8 and ZIP9 may be suppressed to prevent excess cytosolic free Zn^2+^ accumulation, which can be cytotoxic, in response to decreased zinc granular uptake. Of potential importance, ZIP9 functions as an androgen receptor as well as a zinc transporter, and downregulation may further impact β-cell function via associated G-protein signal transduction pathways [41]. 

Inconsistent information is available about ZnT8 and Type 2 Diabetes risk: downregulation is associated with cellular stress and disease development [9,11,12], whereas certain loss-of-function mutations are protective [14]. Lowered ZnT8 expression decreases β-cell/islet zinc content [16,17,18,19]. Consistent with this observation, we demonstrate that ZnT8 haploinsufficient MIN6 cells have reduced total zinc content. ZnT8 haploinsufficiency was not associated with disrupted mRNA expression for any other ZnT paralogues, indicating MIN6 cells do not induce transcription of any *Slc30a* gene to compensate for partial loss of ZnT8, consistent with observations recorded for ZnT8 null mouse islets [19]. ZnT8 haploinsufficiency was associated with lowered mRNA expression for *Slc39a8* and *Slc39a14*, suggesting ZIP8 and ZIP14 are important for coordinating cytosolic zinc influx when ZnT8 transports zinc into granules. Loss of ZIP14 expression in ZnT8 haploinsufficient cells contrasts with our data for transient ZnT8 knockdown, possibly due to differences in zinc requirement following initial disruption. MIN6 cells may downregulate ZIP8 and ZIP14 to prevent destructive effects that could result from cytosolic free Zn^2+^ overload [42] when efflux into secretory granules is reduced. ZIP8 and ZIP14 are phylogenetically closely related [43] and both localise to the plasma membrane and membranes of intracellular zinc-storing organelles and vesicles [33,44]. ZIP8 and ZIP14 transport other metal ions in addition to zinc including non-transferrin bound iron [33,34], cadmium [45] and manganese [35]. Although we did not observe any differences in total cellular iron or manganese levels in ZnT8 haploinsufficient cells, altered transporter expression may induce off-target effects due to redistribution of these metal ions.

### 3.2. Phenotypes of ZnT8 Haploinsufficient MIN6 Cells

An array of endocrine-specific markers function to tightly maintain β-cell phenotype [46]. We have previously demonstrated that multiple transcription factors important for β-cell identity and endocrine function show Zn^2+^-responsive expression (HNF1B, HNF4A, MAFA, MNX-1, NKX2.2, NKX6.1, PAX4, PAX6 and PDX-1) [27]. Since ZnT8 haploinsufficient MIN6 cells exhibit lowered zinc content compared to controls, it is expected that β-cell transcription factor expression and identity is impacted. Accordingly, we observed that ZnT8 haploinsufficiency induced upregulation of *Pax4* and *Hnf4a,* and downregulation of *Foxa1.* Although PAX4 and HNF4A are Zn^2+^-responsive [27,47], their expression is tightly maintained by multiple factors. PAX4 is a major transcriptional regulator of β-cell development, phenotype and function [48], promotes cellular survival by protecting against stress-induced apoptosis [48,49] and prevents β- to α-cell transdifferentiation through inhibiting glucagon expression [50], whereas HNF4A functions to regulate insulin gene expression [51]. Downregulation of FOXA1 additionally has the potential to disrupt β-cell function since FOXA1 acts in combination with FOXA2 to regulate insulin secretion and carbohydrate metabolism [52]. 

Tightly maintained zinc homeostasis is required for cellular signalling, differentiation and proliferation [53]. The changes to transcription factor expression observed in ZnT8 haploinsufficient cells suggest that ZnT8 downregulation impacts β-cell survival and phenotype. We demonstrated that ZnT8 haploinsufficiency lowers proliferation of MIN6 cells and increases apoptosis, possibly due to toxicity resulting from a reduced zinc storage capacity in granules. The lowered proliferation may be promoted by reduced MAPK signalling. MAPK cascades are activated by physiological concentrations of Zn^2+^ [54,55] to regulate gene expression, mitosis, differentiation, metabolism and programmed cell death [56]. The protective effects of reduced ZnT8 expression in vivo may be due to the ability to protect against cytokine cytotoxicity [57], which was not investigated in this study. Interestingly, the two zinc importers that showed changed expression in ZnT8 haploinsufficient cells were ZIP8 and ZIP14, which both have functions in the immune response [58,59]. ZIP14 expression has also been linked to insulin content, processing, and secretion in INS-1E β-cells [60]. Further, since ZnT8 expression is not restricted to β-cells [61,62], polymorphisms may induce effects systemically. The ZnT8 genotype has been associated with differential zinc homeostasis regulation and enhanced inflammatory response upon lipopolysaccharide stimulation in peripheral blood mononuclear cells from patients with Type 2 Diabetes [63]. 

It should be noted that our experiments were carried out in a single population of clonal MIN6 cells and, therefore, the observations may be clone-specific. To further establish the role of ZnT8 haploinsufficiency in β-cell zinc homeostasis and cellular function, further experiments should seek to replicate these findings in a range of genetically diverse clonal populations. Further, rescue experiments should be carried out to confirm the causative role of lowered ZnT8 expression in maintaining cellular function. 

## 4. Materials and Methods 

### 4.1. Cell Line and Culture

MIN6 cells (*Mus musculus*) [64] were cultured in Dulbecco’s Modified Eagle’s Medium containing 25 mM glucose (DMEM, Thermo Fisher Scientific, Waltham, MA, USA), supplemented with 15% (*v*/*v*) fetal bovine serum (FBS, Thermo Fisher Scientific, Waltham, MA, USA), 4 mM L-glutamine, 50 μM β-mercaptoethanol, 100 μg/mL streptomycin and 100 units/mL penicillin (all from Sigma Aldrich, St Louis, MI, USA), at 37 °C in a humidified atmosphere of 95% air and 5% CO_2_. Cells were used from passages 24–38. For characterization of ZnT8 haploinsufficient cells, cells were cultured with growth medium containing either 1 µM (zinc deplete) or 8 μM (normal zinc) of total zinc. To achieve these specified concentrations, FBS was depleted of metal ions through incubation with 5% (*w*/*v*) Chelex-100 (Sigma Aldrich, St Louis, MI, USA) for 1 h, and sterile-filtered through a 0.22 μm syringe filter. Chelex-100- treated FBS was diluted 1:100 in 0.5% (*v*/*v*) nitric acid (HNO_3_) and metal analysis performed by inductively coupled plasma mass spectrometry (ICP-MS; PerkinElmer NexION 350D, Perkin Elmer Ltd, Beaconsfield, UK). Chelex-100–treated FBS was re-constituted with Ca^2+^, K^+^ and Na^+^ to the original concentrations. Chelex-100–treated FBS was added at 15% to DMEM containing 25 mM glucose, 4 mM/L-glutamine, 50 μM β-mercaptoethanol, 100 μg/mL streptomycin and 100 units/mL penicillin, and supplemented with ZnCl_2_ to total Zn^2+^ concentrations of 1 or 8 μM.

### 4.2. Cell Transfection

MIN6 cells were transfected with siRNA targeting *Slc30a8* mRNA (s108999; 5′-3′ sequence: CUUUAAGCCUGACUACAAAtt (Thermo Fisher Scientific, Waltham, MA, USA)) or with Silencer^®^ Select negative control siRNA (4390844; Thermo Fisher Scientific, Waltham, MA, USA) using Lipofectamine RNAiMAX transfection reagent (Thermo Fisher Scientific, Waltham, MA, USA) 24 h after seeding, as per manufacturer’s instructions. 

### 4.3. CRISPR/Cas9 Gene Editing

ZnT8/*Slc30a8* gene editing was carried out using clustered regularly interspaced short palindromic repeats/ CRISPR associated protein 9 (CRISPR/Cas9) gene technology [65]. Cells were transfected with SLC30A8 Double Nickase Plasmid (sc-433687-NIC) or control CRISPR/Cas9 plasmid (sc-418922) (both Santa Cruz Biotechnologies, Santa Cruz, CA, USA) using Lipofectamine 2000 (Thermo Fisher Scientific, Waltham, MA, USA) (Figure S1)**.** At 72 h following transfection, cells were selected using 1.25 ng/μL puromycin dihydrochloride (Santa Cruz Biotechnologies, Santa Cruz, CA, USA) for 6 days and imaged for GFP fluorescence. Cells were transferred into a 96-well plate through serial dilution and clonal cell populations amplified through passaging cells into wells of increasing size. DNA was extracted using the DNeasy Blood and Tissue Kit (Qiagen, Venio, Netherlands). DNA targeted by the CRISPR/Cas9 constructs was amplified by PCR [forward primer: TCATCTCCGTGCTCAAACCC, reverse primer: TCTGTCATCGTGGCACTCAA (Sigma Aldrich, St Louis, MI, USA)] and amplicons separated on 2% (*w*/*v*) agarose gels, supplemented with 0.1% (*v*/*v*) gel red (Biotum, Freemont, CA, USA), at 40 V. Amplicons showing bands shifted from those from control cells (Figure S1) were purified using the QIAquick Gel Extraction Kit (Qiagen, Venio, Netherlands), subjected to a second PCR amplification, treated with ExoSAP-IT PCR Product Clean-up Reagent (Affymetrix, Santa Clara, CA, USA), and Sanger-sequenced at Eurofins Genomics, London, UK. To explore predicted translation of mutated proteins, edited mRNA sequences were uploaded into ExPASy bioinformatics software [66] and amino acid sequences were aligned to native sequences using the Clustal Omega multiple sequence alignment tool [67].

### 4.4. Gene Expression Analysis

Total RNA was extracted from MIN6 cells using TRIzol Reagent (Thermo Fisher Scientific, Waltham, MA, USA), and treated with the high capacity RNA-to-cDNA kit (Thermo Fisher Scientific, Waltham, MA, USA). Primer Blast was used to predict whether primers bind to all *SLC39A* isoforms. PCR plates were loaded using the Biomek FX liquid handling robot (Beckman Coulter, Pasadena, CA, USA) and reactions (0–40 ng cDNA, 0.1 µM UPL probe, 0.2 µM forward primer, 0.2 µM reverse primer and 1X TaqMan Fast Advanced Mastermix (Applied Biosystems, Waltham, MA, USA)) amplified using the Prism7900HT sequence detection system (Applied Biosystems, Waltham, MA, USA) and analysed using sequence detection systems v2.4 software. Assay designs are provided in Table S1. Primer efficiencies ranged from 79 to 120%. The housekeeping gene ubiquitin C (UBC) was used as a control. Differential and relative expressions were calculated using the ΔΔCT method, and statistical significance was calculated using unpaired *t*-tests. Data shown are an average of three biological repeats.

### 4.5. Determination of Cellular Zinc, Iron and Manganese Levels 

Cells were lysed in hot 0.2% (*w*/*v*) sodium dodecyl sulphate (Sigma Aldrich, St Louis, MI, USA). Samples were diluted 1:100 with trace element grade 0.5% nitric acid (Sigma Aldrich, St Louis, MI, USA) and ICP-MS was used to establish zinc concentrations. The total zinc, iron and manganese contents were expressed relative to total protein, which was determined through the Bradford assay (Bio-Rad, Hercules, CA, USA). Unpaired *t*-tests were used to calculate the statistical significance of differences in cellular zinc, iron and manganese levels.

### 4.6. Cellular Proliferation and Apoptosis 

The rate of cellular proliferation was assessed using the Cell Proliferation ELISA, BrdU (colorimetric) assay (Sigma Aldrich (Roche), St Louis, MI, USA). A 2 h incubation period with BrdU labelling solution was used. The rate of cellular apoptosis was assessed using Caspase-Glo^®^ 3/7 assays (Thermo Fisher Scientific, Waltham, MA, USA), using 50 μL reagent per 100 μL sample. 

### 4.7. Immunoblotting

Protein lysates (20 µM) were prepared in 2× loading dye (Thermo Fisher Scientific, Waltham, MA, USA), heated (10 min, 72 °C), and separated on 12% acrylamide SDS-PAGE gels (Thermo Fisher Scientific, Waltham, MA, USA) supplemented with 0.1% (*w*/*v*) SDS (200 V, 40 min), and transferred onto nitrocellulose membranes [GE Healthcare Life Sciences Amersham, UK] (1 h, 100 V). Membranes were blocked with 5% (*w*/*v*) BSA/Tris-buffered saline [TBS (50 mM Tris-HCl pH 7.5, 150 mM NaCl)] and 0.05% (*v*/*v*) TWEEN-20 [TBST (1 h, room temperature], and incubated with primary antibodies diluted in 1% BSA/TBST [ERK1/2 (#9102, Cell Signalling Technology, Danvers, MA, USA, 1:1000), p42/44 ERK1/2 (#4370, Cell Signalling Technology, Danvers, MA, USA, 1:2000), RPS6 (#2217, Cell Signalling Technologies, Danvers, MA, USA, 1:1000), and Ser235/236 RPS6 (#4858, Cell Signalling Technologies, Danvers, MA, USA, 1:2000)] (16 h, 4 °C), followed by HRP-linked secondary antibodies diluted in 1% BSA/TBST (NA934V, GE Healthcare Life Sciences Amersham, UK, 1:15,000) (1 h, room temperature). Membranes were visualised following treatment with ECL Western Blotting Detection Reagent (GE Healthcare Life Sciences, Amersham, UK). For detection of additional proteins, membranes were treated with stripping buffer (Thermo Fisher Scientific, Waltham, MA, USA), as per manufacturer’s instructions. Band intensities were analysed using ImageJ software and phosphorylation of ERK1/2 and RPS6 were expressed as a ratio to total ERK1/2 or RPS6.

### 4.8. Insulin Secretion Assays

MIN6 cells were starved in DMEM containing 3 mM glucose (24 h, 37 °C). Cells were washed with PBS, incubated in Krebs-Ringer bicarbonate buffer (KRBH, 10 mM Hepes, 2 mM NaHCO_3,_ 70 mM NaCl, 3.6 mM KCl, 0.5 mM NaH_2_PO_4_, 0.5 mM MgSO_4,_ 1.5 mM CaCl_2,_ 10 mM NaCl, 0.1% BSA, adjusted to pH 7.4 with NaOH) (30 min, 37 °C), supplemented with 3 mM glucose (30 min, 37 °C), followed by KRBH supplemented with 3 mM glucose, or 3 mM glucose and 40 mM KCl (30 min, 37 °C). The supernatant containing secreted insulin was aspirated and diluted 1:20. The amounts of secreted insulin were determined using the colorimetric Rat/Mouse Insulin ELISA assay kit (EZRMI-13K, Merck Millipore, Burlington, MA, USA), following manufacturer’s instructions, and normalised to total protein, determined through Bradford assays (Bio-Rad, Hercules, CA, USA).

## 5. Conclusions

We explored the impact of lowered ZnT8 expression on the phenotype of MIN6 cells only during zinc depletion. Diminished ZnT8 abundance downregulated ZIP8 and ZIP14 expression, decreased zinc accumulation, altered expression of key β-cell transcription factors and reduced cellular survival via MAPK signalling cascades in MIN6 cells, consistent with the effects of stress-induced ZnT8 suppression [9,11,12]. Overall, we demonstrate coordinated ZIP-ZnT8 zinc trafficking is important for β-cell zinc metabolism and that ZnT8 haploinsufficiency impacts β-cell phenotype and reduces cellular survival. The results suggest that protective effects of ZnT8 loss-of-function mutations are either different between mouse and human or are manifested at the organismal level rather than in cell culture.

## Supplementary Materials

Supplementary materials can be found at https://www.mdpi.com/1422-0067/20/21/5485/s1.
