# Supplementary material for: ZnT8 Haploinsufficiency Impacts MIN6 Cell Zinc Content and β-Cell Phenotype via ZIP-ZnT8 Coregulation
Source: Int J Mol Sci. 2019 Nov 4;20(21):5485. doi: 10.3390/ijms20215485 (PMC6861948; doi:10.3390/ijms20215485)
Supplement: Supplementary file 1 [file ijms-20-05485-s001.zip › Figure S2.pdf]

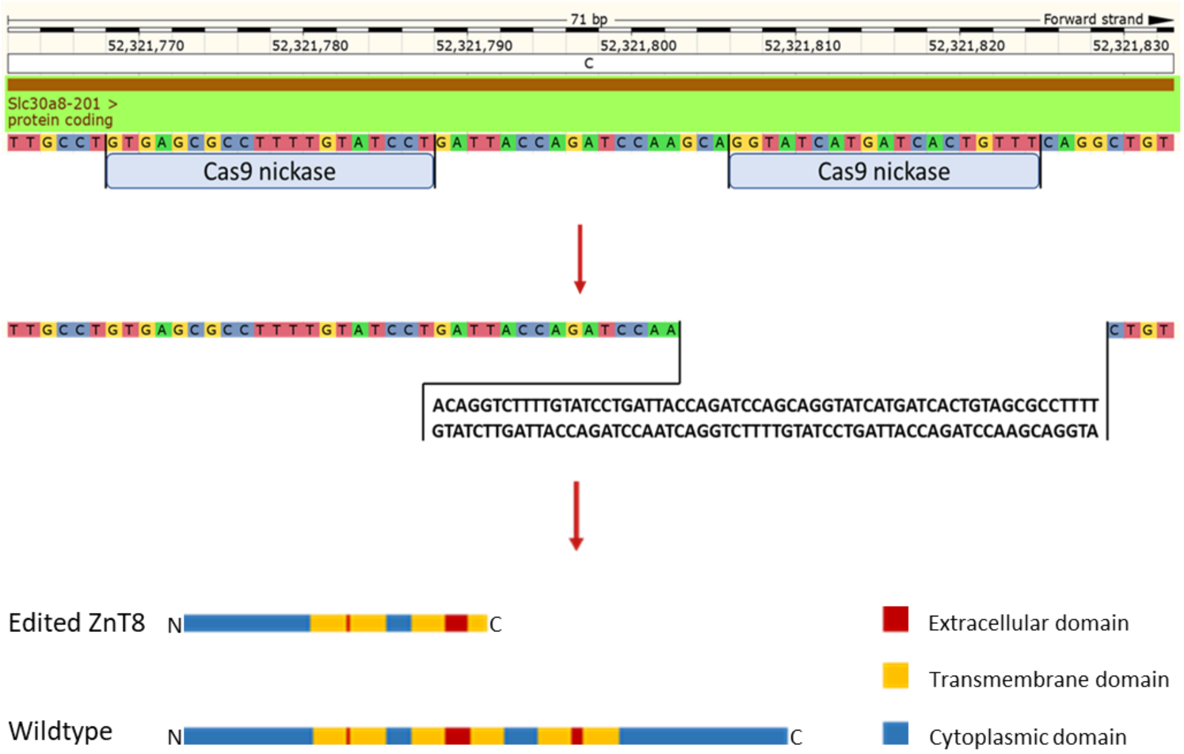
71 bp
Forward strand
52,321,770
52,321,780
52,321,790
52,321,800
52,321,810
52,321,820
52,321,830
C
Slc30a8-201 >
protein coding
TTGCCTGTGAGCGCCTTTTGTATCCTGATTACCAGATCCAAGCAGGTATCATGATCACTGTTTCAGGCTGT
Cas9 nickase
Cas9 nickase
TTGCCTGTGAGCGCCTTTTGTATCCTGATTACCAGATCCAA
CTGT
ACAGGTCTTTTGTATCCTGATTACCAGATCCAGCAGGTATCATGATCACTGTAGCGCCTTTT
GTATCTTGATTACCAGATCCAATCAGGTCTTTTGTATCCTGATTACCAGATCCAAGCAGGTA
Edited ZnT8
N
C
Wildtype
N
C
Extracellular domain
Transmembrane domain
Cytoplasmic domain
