# Supplementary figures and images for: ZnT8 Haploinsufficiency Impacts MIN6 Cell Zinc Content and β-Cell Phenotype via ZIP-ZnT8 Coregulation
Source: Int J Mol Sci. 2019 Nov 4;20(21):5485. doi: 10.3390/ijms20215485 (PMC6861948; doi:10.3390/ijms20215485)

A

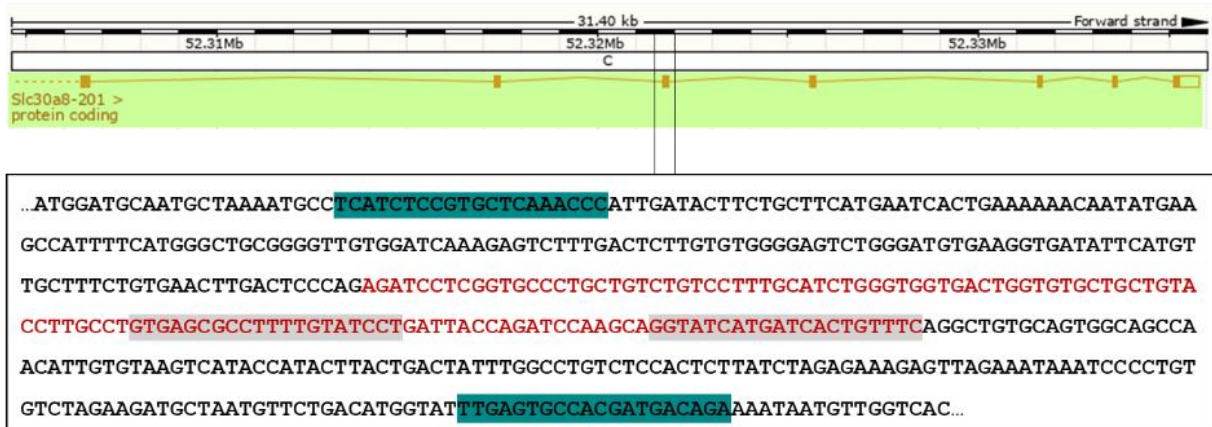

B

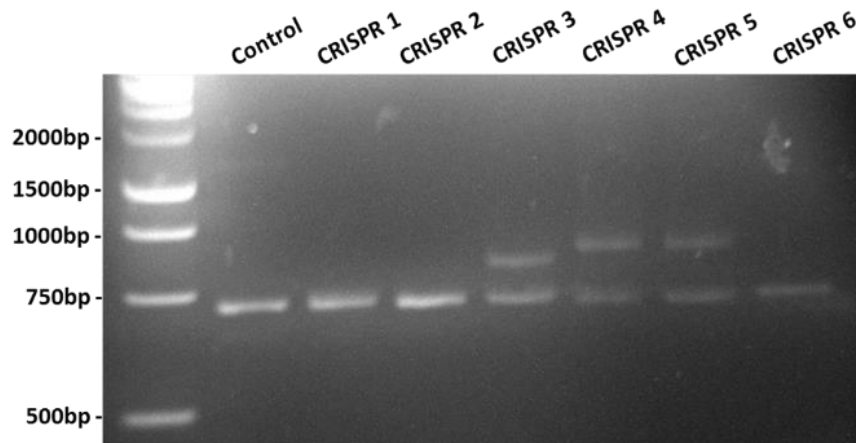

Supplement: Supplementary file 1 [file ijms-20-05485-s001.zip › Figure S1.pdf]
